# Experiences of externalisation in recovery from Anorexia Nervosa: a reflexive thematic analysis

**DOI:** 10.1186/s40337-024-01087-9

**Published:** 2024-10-07

**Authors:** Sophie Cripps, Matthew Pugh, Lucy Serpell

**Affiliations:** Research Department of Clinical, Educational and Health Psychology, University College London, Gower Street, London, WC1E 6BT UK

**Keywords:** Anorexia Nervosa, Anorexia voice, Eating disorder, Externalisation, Recovery, Qualitative, Thematic analysis

## Abstract

**Background:**

Many individuals perceive Anorexia Nervosa (AN) as a part of their personal identity. Externalisation of the problem is a practice that is often taken up within NICE recommended treatments for AN. Dominant understandings of externalisation are that this practice involves making the “problem” a separate entity, external to the individual. It is an attitude taken by the client and family, stimulated by the therapist to build engagement with treatment and supportive relationships around the individual. However, there is a paucity of research exploring the therapeutic effects of this approach. This research aims to address this gap by exploring the role of externalisation in treatment for AN to elicit an understanding of how this practice is experienced including how it can help and hinder recovery.

**Methods:**

Thirteen adults with a current and/or past diagnosis of AN participated in semi-structured interviews. This qualitative study used a reflexive thematic analysis.

**Analysis:**

Participants described their experience of externalisation as a journey which is depicted by four main themes. ‘Separating the AN from the self’ reflects the tensions inherent in learning to distinguish between one’s perceived sense of "self" and "the AN". ‘Making sense of the AN’ describes the experience of language forms used to separate the AN from the individual’s identity. ‘Feeling seen, or unseen as a person beyond the AN’ illustrates the helpful and harmful effects of externalising practices on relationships. ‘Navigating a complex relationship with the AN’ depicts the effects of one-, versus two-way externalisation on the individual’s relationship to AN.

**Conclusions:**

The notion of separating one’s internal dialogue from one’s concept of self may initially be rejected by the individual experiencing AN. However, as the individual develops trust in the therapist and becomes socialised to common forms of externalising language, they may begin to realise two sides within them, a perceived “healthy self” and “the anorexia voice”. However, social-cultural discourses around eating contribute to ambiguity during the differentiation between these two voices, thus elucidating the effects of an absence of problem deconstruction alongside externalisation within ED-focussed treatments. Externalising practices were most helpful when led by the individual using their own experience-near language and least helpful when they did not permit the individual to feel seen as a person beyond the AN. Therapists, treatment teams and family members should be cognisant of the emotional effects of language used to externalise AN. Importantly, they should ensure that externalisation is practiced within the spirit of narrative therapy from which it originates.

**Supplementary Information:**

The online version contains supplementary material available at 10.1186/s40337-024-01087-9.

## Abstract (plain language summary)

This research explores the experiences of individuals with current and/ or past experience of anorexia nervosa (AN) who received NICE approved psychological therapies in which their eating disorder (ED) was separated from their identity through externalisation. Within these therapies, externalisation is a therapeutic practice that involves viewing AN as an external entity or illness that is separate from the identity of the individual who is experiencing AN. Participants described how it was initially difficult to accept that their thoughts, feelings and behaviours were influenced by something external to them. However, over time, the individual experiencing AN began to trust in this idea which was proposed to them throughout treatment. This perception of their experiences had both positive and negative effects on their recovery. This research suggests that individuals experiencing AN, their therapists, treatment teams and family members should be curious about the emotional effects of the language forms used to separate AN from the person’s identity. Language which empowered individuals in relation to their eating difficulties and which permitted individuals to feel seen, heard, and validated as a person beyond AN supported their recovery. The findings underpin the importance of individual-led externalisation and thus, practicing externalisation with adherence to the core narrative therapy principles which underlie this therapeutic practice. In doing so, externalisation may be used most helpfully to support recovery from AN.

**Supplementary Information:**

The online version contains supplementary material available at 10.1186/s40337-024-01087-9.

## Background

### From "Anorexic" to a "person with a lived experience of Anorexia Nervosa"

The way that Anorexia Nervosa (AN) is talked about has changed over time; there has been a shift from talking about the person as "anorexic" to a "person with a lived experience of AN" [1]. Key in this shift is the spirit of externalisation which is a practice borrowed from narrative therapy that is utilised in National Institute of Care Excellence approved therapies for AN to support people's recovery [2]. Externalisation refers to the linguistic separation of the problem from the person; it is an approach that fosters efforts to move away from pathologizing discourses which totalise a person as ‘disordered’, to viewing human problems as arising from and being maintained by oppressive stories which dominate the person’s life [3]. Externalising practices create a context in which problems are treated as separate from people; this is a unique process within a Western culture that values a scientific medical model that situates problems within individuals’ biology and character and contributes to people’s internalisation of the problem [4]. When a problem is internalised, people assume the problem is a facet of their nature or inner self and thus it becomes fused with the person’s identity [4].

### "Recovery" from Anorexia Nervosa

There is currently a lack of consensus regarding the definition of recovery in treatment for AN [5]. However, individuals with lived experience typically reject the definition of recovery as an 'end-point' on reaching a specific body-weight, instead, they highlight the importance of emotional coping and life quality, viewing recovery as a 'process' of reconnecting, rediscovering and rebuilding a previously neglected or lost part of the self and therefore, a journey of reclaiming personal identity beyond that of the ED identity [6–10]. AN typically emerges during adolescence and tends towards a protracted course [11]. Treatment often succeeds at restoring weight; however, it is common for ED cognitions and behaviours to persist [12]. Accordingly, further research to increase treatment efficacy is required and within this, given the complexities associated with recovery from AN, exploration of how therapeutic interventions can assist individuals in navigating issues of identity is warranted.

### Externalisation in treatment for Anorexia Nervosa

Externalisation is a key tenet of Family Based Treatments [13, 14], where family members are encouraged to separate the individual from AN through language and metaphor, AN is viewed as an ‘unwanted temporary illness’ which threatens to take the individual's life [15]. Sometimes, the externalised ED is given a name and personified, creating a separate entity [16]. This approach is thought to preserve the relationships between the individual, their family and treatment team through reducing negative interactions, increasing compassion and united support [17]. Externalisation is also often used within individual treatments, including Cognitive Behavioural Therapy for Eating Disorders [18], Adolescent Focussed Psychotherapy [19, 20] Maudsley Model of Anorexia Nervosa Treatment for Adults [21] and Focal Psychodynamic Therapy [22].

Externalisation is applied in different ways and for different purposes across treatment models. Within the aforementioned NICE approved treatments for AN, externalisation is most commonly viewed as a therapeutic strategy or technique to effect behavioural change through placing emphasis on encouraging the individual and their family or close others to view the experience of eating difficulties as being attributable to the influence of a powerful external force over the individual [13]. In contrast, within narrative therapy, where externalisation originated, it is positioned not merely as a technique but embedded within the broader philosophical narrative worldview where the person is positioned as the expert in their life [23]. Within narrative therapy, the use of metaphor fosters a different way of seeing AN rather than offering a "literal truth" [24, 25].

### Externalisation as a practice from narrative therapy

Externalisation is a language practice developed by Michael White, a co-founder of narrative therapy [26]. Within narrative therapy, externalising refers to a frame of mind and deliberate method of discourse (communication) that invites people to think about their lives and problems in unique ways [4]. It is an attitude and orientation in conversations where the ways that therapists engage the person (and their family) in talk, linguistically separate the person from the problem [3]. The narrative stance is situated in a social constructionist framework; i.e., that our sense of what is real in the world and who we are is constructed by the language we use and the stories made possible through the use of this language [4]. Thus, externalising conversations represent the therapeutic stance which maintains a constant discourse that views people as distinct from their socially constructed identities, sense of reality and problems [4].

Narrative therapists see, hear and think about problems as being shaped and given meaning by narratives; problems are not viewed as hard realities that permanently define people, rather they are viewed as ‘problem stories’ through which people know themselves and are known by DeKruyf [27]. The spirit that informs externalisation in narrative therapy is that the person is not the problem, the problem is the problem, and the person is assumed to have beliefs, competencies and commitments to assist them in changing their relationship with the problem [28]. The separation of the problem from the person creates space for seeing the problem and thinking about it in new ways, opening possibility for developing a different relationship with ones’ self and one’s life stories [27]. The resulting effect, when externalising practices are careful and thoughtful, is that the individual feels less oppressed and more empowered in relation to the problem [29].

Externalising is intended to shift the focus of conversation onto the relationship between the person and the problem as opposed to a singular focus on a ‘problem-person’ [4]. Relative-influence questioning is a particular style of questioning that is used to help clients begin to view their problems as separate from themselves [3]. Central to this style of questioning is inquiring about how the problem has been affecting the person's life and relationships. In this way, clients are invited to think of themselves not as problem-people but as individuals who have a relationship with a problem. Asking questions in a way that assumes the problem and person are separate helps clients to externalise the problem and internalise personal agency [3]. This type of questioning also opens up the possibility that clients may report that on some occasions the problem influences them to the point of oppression, whereas on others, they may resist the problem [3]. The latter refers to experiences or events that are exceptions to the problem-saturated narrative that have dominated the individual’s life and identity. These ‘unique outcomes’ are the stepping stones from which lives may be re-authored [3].

The narrative approach rests on the assumption that narratives constitute identities, lives and problems [30]. According to this position, the process of therapeutic re-authoring personal narratives changes lives, problems and identities because personal narratives are constitutive of identity. Within a narrative worldview, a person's identity is understood as multi-storied whereby their relationship with the problem is one of the many stories of their life; however, when a problem narrative dominates (i.e., the AN identity story), other stories of self are obscured [31]. Not only do these dominant narratives describe a person to themselves and to others, the individual also performs their meaning, therefore, they shape a person's reality [25]. Developing therapeutic solutions to problems within the narrative frame involves opening space for the authoring of alternative stories, the possibility of which have previously been marginalised by the dominant oppressive narrative which maintains the problem [3].

In narrative therapy, externalising conversations are guided by maps that involve the following intertwined and interdependent processes: (1) deconstruction of the problem, unpacking the meanings ascribed to the problem and inviting an experience-near name for it; (2) mapping, evaluating and reflecting on the problem’s influence, as well as the person’s influence over the problem (relative influence questioning); (3) identifying a preferred story through the exploration of what is important to the individual; and subsequently, (4) creating foundations for action to move away from the problem’s influence and towards the preferred identity [29]. Through engaging the person in these steps, unique outcomes or identity stories that have been hidden by the problem stories of their life are generated through conversation. As unique outcomes are generated, therapists listen out for and invite the person to thicken the meaning of identity stories that were previously hidden. As these stories are thickened, their meaning becomes performed as they align their actions with preferred identity stories, including valued senses of self [29].

### Michael White’s final writing on externalising Anorexia Nervosa

Within Michael White's final writing regarding his perspective on externalisation in treatments for AN "Continuing the Conversation: On Anorexia", he described key considerations for externalising conversations [31]. Firstly, he discussed how the aim of early externalising conversations should be to develop a rich characterisation of the problem with attention to supporting the generation of experience-near definitions using the client's own words, thus recognising that individuals do not have identical experiences of AN. He discussed how inquiry that contributes to person-led characterisation of problems encourages people to orient themselves to the problem as "investigative reporters”, a stance that supports the individual to develop an understanding of the consequences of AN, its operations, activities and agenda for their life. In the further development of this investigative reporter role, people can be encouraged to develop an "expose" of the contexts of life that reinforce AN, hence acknowledging the influence of social context.

White also addressed the importance of avoiding the imposition of metaphors to describe the person's relationship with AN, stating that externalising conversations should not introduce a ready-made characterisation of a problem, or the person's relationship with it. Further, that given the significance of these metaphors in shaping people's experience and actions, their effects on the person should be monitored. Importantly, he suggested that the metaphors people take up to characterise their relationship with the problem can have significant influence on the intensity of people's relationship with the problem. White acknowledged the intensity of people's relationship with their body, eating-related experience and emotion in AN; he indicated that battle and contest metaphors can exacerbate an already intense relationship, foster a "hot engagement" with the problem and contribute to AN becoming an "overpowering adversary". White suggested that metaphors which foster a less intense engagement with the problem are more conducive to the further development of the ‘investigative reporter’ posture that supports a more detached relationship with the problem. White explained that this posture makes it possible for people to make decisions about how they may take steps to reclaim their lives, or to undermine the influence of AN.

White discussed that whilst individuals can use metaphors that evoke battle, conquest or fight for survival to define their relationship with AN, therapists should be mindful of their hazards which are cause for ethical concerns such as the effect of increasing the intensity of relationship between the individual and the problem, and the risk of contributing to a sense of hopelessness, personal failure and inadequacy when attempts to "vanquish AN" do not eventuate. He suggested that the further development of these metaphors within the context of therapeutic conversations could result in a fortress type mentality, exacerbating a sense vulnerability and isolation, diminishing personal agency. Consequently, he advised that whilst an individual's use of such metaphors to characterise their relationship with AN can be honoured, therapists must avoid contributing to their further development and should thus be attentive to other less hazardous metaphors present in conversation.

Lastly, White explained how there will be aspects of AN that the person appreciates and these should be acknowledged and explored within therapeutic conversations; stating that rarely will the individual take a total position in relation to a problem. Therefore, inviting the individual to take a position that is total (all for AN, or all against it) contributes to anxiety and apprehension to take steps towards challenging the problem. He suggested that the therapist's openness to what is valued about aspects of AN can lead to rich externalising conversations. Further, that learning what an individual gives value to and tracing the social and relational history of these valued aspects of life creates a foundation for collaboration and can support expressions of life that are not in harmony with the problem.

### Research on externalisation of Anorexia Nervosa

Metaphorical themes used to externalise AN in NICE approved treatments are dualistic (e.g., AN as an illness), militaristic (e.g., AN as a terrorist), satanic (e.g., AN as a demon) or animalistic (AN as a parasite) [32]. ‘Countering That Which Is Called Anorexia’ is one of the first narrative writings describing how externalisation can be used in treatment for AN [15]. The authors suggested that AN has a voice of its own, which acts as a ‘discursive parasite’. Further, that once separated from the person, they can be helped to find alternative discourse resources that assist them in gaining power to resist ‘the parasitic voice’. Therefore, externalisation of AN sometimes takes the form of an internal ‘voice’ [33]. However, research has questioned whether experiences of ED voices are a consequence of externalisation, or rather a discrete perceptual experience that individuals are not socialised into [34]. Popular terms for the externalised entity include the “Anorexia/Eating Disorder/Sick Self or Voice”, “They/It”; these are contrasted with terms such as the individual's "Real/True Self", "Healthy Self/Voice" or their whole identity "You/Me/I" [32]. Within treatments for AN, these terms will be used in externalising conversations to engage individuals in the treatment and recovery process. They may also be used to engage individuals in therapeutic exercises such as writing letters to AN as their "friend" or "foe" [21, 35].

Whilst individuals can experience externalisation as a helpful aspect of treatment [36], research suggests that externalisation can have unintended effects. For instance, individuals can feel as though they are being wrongly accused of being dishonest, or not taken seriously when all their behaviour is labelled as part of AN [37]. Moreover, externalisation can be experienced as more or less helpful at different stages of treatment, depending on how individuals perceive the relation between their identity and AN [37]. For example, individuals who perceive AN as a core, valued part of their identify may feel invalidated when told to consider their own actions are merely ‘the Anorexia’ [38]. Additionally, some parents feel that externalisation can exclude the individual’s voice in treatment, and when confined to an illness or other adversarial metaphor, place them and their family at risk of exhaustion and reduced agency [39]. Young people too can feel that with externalisation, their voice can become lost in treatment as it is assumed to be the voice of the illness/AN by parents and/or their therapist, thereby externalising their identities with the AN [40].

Individuals with lived experience of severe and enduring AN reject medical discourse of recovery which confines them to an illness metaphor as it conveys an endpoint where the illness has gone [6]. Such medical discourse limits opportunities for individuals to ascribe meaningful associations to their shifts in relationship with AN overtime and excludes them from the category of recovery. Instead, they construct their experience of ‘recovery' as a journey of re-claiming their voice and life from AN. Such research has demonstrated how individuals with lived experience take up relational metaphors to describe their experience of recovery as they are better able to capture their complex social realities in which AN is experienced as both a part of them, as well as an external entity that they feel in relationship to [6, 7].

Within treatments for AN, clinical-medical "illness" and "battle" metaphors which are adversarial, hierarchical and linear permeate the clinical field and shape our understandings and therapeutic practices. These metaphors can neglect the experiencing person's reality in which they can continue with significant suffering under the influence of AN [6, 7]. Therefore, it has been proposed that relational, flexible and non-linear metaphors are better able to describe how individuals navigate an ongoing relationship with AN rather than its elimination [6, 7]. Such research has suggested a need to re-examine the effects of dominant metaphors in treatments for AN.

FBT/FT-AN therapists suggest that externalisation can support recovery when used in conjunction with other skills including listening, rapport building, effective timing of interventions, engaging with a family and understanding their relationship dynamics, as well as knowledge of barriers to externalisation including problem awareness, age and illness duration [41]. A systematic review of narrative therapy treatment components for the treatment of EDs found that the majority of articles spoke of externalisation as a therapeutic technique rather than positioning it within the broader narrative worldview where the person is positioned as the expert of their life [42]. It is possible that practicing externalisation within the broader narrative worldview may increase its effectiveness at supporting recovery from AN.

### Study aims and rationale

Externalisation is practiced and experienced differently within NICE approved ED interventions compared to the intention of this practice within narrative therapy where it was originally proposed. Little is known about people’s lived experiences of externalisation within NICE approved treatments for AN. Whilst research has revealed negative counter-effects of externalisation within inpatient treatment [37], research exploring the perspective of individuals who have completed treatment(s) within a range of settings is lacking. Such research would provide insights into how externalisation helped and hindered across the entire recovery process, whilst also considering the influence of treatment context. This research aimed to address this gap by examining the experiences of adults who have and have not recovered from AN following evidenced-based treatment. Within the overarching aim of exploring how externalisation effects people’s experience of NICE recommended treatment(s) for AN, we hoped to elicit an understanding how and why externalisation may help and/or hinder the process and maintenance of recovery, including its effects on peoples’ relationships with themselves and others.

## Methods

### Design

A reflexive thematic analysis (RTA) [43] was used to derive themes to explain how externalisation can help and hinder recovery, allowing rich and descriptive insights into people’s experiences of externalisation during and beyond treatment(s) for AN to be obtained.

### Data collection

The first author developed a semi-structured interview schedule utilising their personal and professional experiences, key literature and supervision. It focussed on: the overall experience of externalisation during treatment and its influence on relationships, engagement, change, recovery and relapse (see Additional file 1).

### Ethics

This study received ethical approval from UCL Research Ethics Committee (approval number 22063/001). Prior to partaking in an interview, participants submitted their signed consent and were made aware of their right to withdraw, skip questions, and take breaks as required. Participants were debriefed and sent a support card following their interview.

### Recruitment

Purposive sampling was used to identify UK residents aged 16 and above, with a current and/or past diagnosis of AN for which they had completed one or more NICE recommended psychological treatments. Individuals did not meet criteria if they were not familiar with externalisation, they had received treatment over 5 years ago, they did not understand spoken or written English, or they were receiving inpatient treatment for AN at the time of data collection. Recruitment was conducted online through social media.

### Procedure

Individuals who were interested in participating were provided a participant information sheet (PIS) and completed a study screening questionnaire. Eligible participants completed a pre-interview questionnaire which contained the consent form and Eating Disorder Examination Questionnaire (EDE-Q 6.0); a 28-item self-report measure of ED symptoms which generates a global score indicating ED severity [44]. Data collected through the screening questionnaire and pre-interview survey provided relevant background information to describe the sample (Table 1). Participants then took part in an online interview which lasted 40 to 90 min.

**Table 1:** Participant information

Participant number (P)	Age	Gender	Ethnicity	Treatment(s) received	Time since completed most recent treatment	Current or past AN diagnosis	Subjective recovery status
1	20	Female	White British	CBT-E	Under 1 year	Past	In recovery
2	32	Female	White British	CBT-E, FBT, MANTRA, FPT	4 years	Current	In recovery
3	32	Female	White British	CBT-E, FPT	1 year	Past	In recovery
4	30	Female	White British	CBT-E	3 years	Past	In recovery
5	31	Female	White British	CBT-E, MANTRA, FPT	Under 1 year	Past	In recovery
6	26	Male	White British	FBT, CBT-E, MANTRA	2 years	Current	In recovery
7	22	Female	White British	CBT-E	2 years	Past	In recovery
8	24	Female	White British	CBT-E, MANTRA	2 years	Current	In recovery
9	28	Female	White British	CBT-E	Under 1 year	Past	In recovery
10	24	Female	White British	FT-AN, CBT-E, MANTRA	Under 1 year	Current	In recovery
11	39	Female	White British	CBT-E, MANTRA	In treatment	Current	In recovery
12	24	Female	White British	CBT-E	2 years	Past	In recovery
13	29	Female	White British	FBT, CBT-E, MANTRA	Under 1 year	Current	In recovery

CBT-E = Cognitive behavioural therapy for eating disorders

FPT = Focal psychodynamic therapy

MANTRA = Maudsley model of anorexia nervosa treatment for adults

FBT = Family based treatment

FT-AN = Family therapy for Anorexia Nervosa

### Participants

All participants were White British, aged between 20 to 39 (mean = 27.8) and identified as being ‘in recovery’, whereby they were continuing to experience ED symptoms but were actively managing these to sustain their recovery. The majority of participants reported to have initially received a diagnosis of AN within adolescence following which they had completed multiple NICE recommended treatments across inpatient and outpatient settings. The mean global EDE-Q score was 2.81 (range = 3.84, SD = 1.38), which sits between normative and clinical ranges [45].

### Data analysis

The first-author (SC) employed an interpretive paradigm to RTA whereby they explored meanings, relations, nuances, contradictions and variations in people’s experiences whilst holding reflexive awareness of their own influence. Both inductive and deductive approaches to coding and analysis were adopted; theoretical, research and experiential knowledge was drawn on to develop the research question and interview, however open coding was utilised to understand and emphasise participant meanings. The literature review and the researcher's professional and personal experiences informed their thinking about how externalisation may help and hinder ED recovery and therefore what questions to include within the interview schedule (deductive). However, coding and theme development were directed by the content of the data and themes were progressively refined in light of the data content (inductive).

SC watched each recorded interview whilst reading the corresponding transcript, noting reflections. They then worked systematically through the dataset whereby data segments were given analytically-meaningful descriptions. Subsequently, code labels were collated and data segments for each code were compiled. Shared patterns of meaning were then identified whereby clusters of codes sharing a core concept were compiled as themes. Thereafter, themes were assessed as to whether they highlighted the most salient patterns of meaning and were reviewed for their core concept and fit into the data’s overall story. These themes were reviewed by the research supervisors (MP and LS); their perspectives on the generated themes were subsequently discussed within supervision. Lastly, the themes were named and given a synopsis whereby analytic narrative and data extracts were weaved together to address the research question.

### Reflexivity

Throughout the research, SC, the first author (I) reflected on my position as a white British heterosexual female trainee clinical psychologist with personal and professional experience of externalisation in treatments for AN. The process of keeping a reflexive journal and engaging in reflexive discussion in supervision helped to maintain clarity of thought about the topic and to hold an open, curious mind with an awareness of one’s own pre-conceived ideas. However, in line with the RTA approach, I utilised my subjectivity as a tool to delve deeper into the data and extrapolate meanings further.

Through personal and professional experience, I had observed how individuals could experience fear and vulnerability in relation to what was externalised within treatment. Consequently, I was curious to learn whether dominant language forms used to externalise AN contributed to feelings of entrapment and powerlessness. I therefore approached data collection and analysis with interest in the emotional and relational effects of externalising language. Accordingly, focus was placed on the role of language in serving to empower versus disempower individuals in relation to their experience of eating-related difficulties.

MP is a white British heterosexual clinical psychologist with lived experience of AN and clinical experience working with individuals given a diagnosis of AN. He has a research and clinical interest in internal eating disorder voices. LS is a female heterosexual clinical psychologist active in research into eating disorders as well as seeing individuals with eating disorders clinically. Both MP and LS have experience using externalisation as part of their therapeutic work. As supervising researchers, MP and LS recognised that individuals given a diagnosis of AN could experience externalisation as both helpful and harmful, depending on the way it is used by professionals.

## Results

The themes and subthemes are shown in Table 2.

**Table 2:** Themes and subthemes developed during the analysis

Theme	Subtheme
1. Separating Anorexia from the self	(1a) It was my voice, it was me
	(1b) A realisation of self as a person beyond Anorexia
	(1c) Are they my thoughts, or are they Anorexia’s?
2. Making sense of the Anorexia	(2a) Person-led, experience-near language
	(2b) Creative means to express complex feelings
3. Feeling seen, or unseen as a person beyond the Anorexia	(3a) Replacing trust in the Anorexia with trust in the therapist
	(3b) Scaffolding supportive relationships
4. Navigating a complex relationship with the Anorexia	(4a) A constant presence; to listen, or not to listen?
	(4b) Reclaiming a valued sense of self

**1.**: **Separating Anorexia from the self**

Treatment sought to separate the ED from the individual’s sense of self. Participants conveyed the tensions inherent in learning to distinguish between one’s sense of “self” and “the anorexia”, including the influence of the therapist in this process and the impact of diet culture.

***1a.***: ***It was my voice, it was me***

Participants reflected on initially experiencing no separation between their sense of “self” and “the anorexia”. Consequently, they did not feel understood when others used externalising language; this incongruence negatively impacted on their trust in treatment:

“When it was first introduced, I didn't understand it, it affected the relationships because I felt like the people who were treating me didn't know what they were talking about, so I had no confidence in them” (P2).

It was difficult to comprehend the notion of an external entity with its’ own voice exerting influence over them. Hence, externalisation initially evoked “scepticism”, “confusion”, and increased resistance. However, later on in recovery, participants realised “two sides” within them; one that wanted “comfort” and “safety” from the anorexia, and one that wanted a “normal life”:

“I really couldn't get my head around it as a young person, but I think as an adult it definitely became more useful and a bit more appropriate. I started to find it helpful in terms of maybe seeing the two separate sides of me” (P5).

Participants emphasised that others should “wait until the person is ready” to be “receptive” to externalising their internal experiences*.* Some highlighted the therapeutic benefit of the therapist initially exploring what led to the person being in treatment, as well as efforts to build rapport “with the person”. These conversations led to their “own conclusions” about the problem’s influence on their lives.

***1b.***: ***A realisation of self as a person beyond the Anorexia***

In treatment, therapists would engage participants in conversations that differentiated between their “healthy self” and “the anorexia”:

“We have discussions about whether I thought it was my healthy self or my eating disorder self that was making each decision” (P7).

As treatment progressed, participants began to accept the notion that their thoughts, feelings and behaviours were under the influence of what felt like an “external force”. Engagement with this concept helped to promote engagement in treatment:

“I now refer to it as ‘the’ eating disorder because it's not my problem…a problem with my personality, it's not a part of me. It’s just an illness. And as it’s not a part of me, I feel I can treat it better; that helped me to recover” (P1).

The illness metaphor alleviated the perceived permanence of anorexia, deterring internalisation of the problem, which increased self-compassion, validation, and hope. In this way, externalisation contributed to the sense that there was more to the person than the anorexia, thus creating opportunities to re-author a sense of self that had been obscured by the dominant anorexia identity:

“I needed to be reminded that I was a person beyond this. I'd forgotten…I didn't have many memories of who I was before anorexia, so it was very helpful in reminding me that I wasn't just an illness” (P2).

The linguistic separation of self from the AN created opportunity to shift their relationship with AN. Consequently, it became possible to resist the influence of AN on their life. Participants were able to “think more clearly” and access “a rational self”. They began to identify “anorexia thoughts” or “the anorexia voice” (both referred to hence forth as the AV) and separate these from thoughts perceived as generated by their “healthy self”.

“It gave me a bit of direction because it gave me something specific to challenge rather than it just being thoughts in my head” (P7).

Their difficulties became “more practical”, “less emotional” and “more manageable”, enabling some participants to become more attuned to their needs. Implicit within their narratives was that externalising the problem internalised personal agency:

“Once you separate, it makes it easier to not do the things that it’s [the AV] telling me to do, because I feel like I can say no to it if it’s not my thought…like ‘I actually don't want to do that’” (P4).

***1c.***: ***Are they my thoughts, or are they the Anorexia's?***

Despite the linguistic separation between a sense of one’s “healthy self” and “the anorexia”, participants emphasised how difficult it was to distinguish between these two voices*.* In reference to this dilemma, participants highlighted the influence of dominant discourses such as diet culture. Consequently, thoughts which appeared “normal” within their social-cultural context were described as “grey areas” evoking uncertainty as to whether the voice of one’s perceived “healthy self” was actually the AV “in disguise”:

“‘Don't eat that because if you eat too much sugar, you’ll get diabetes’…those kinds of thoughts were harder to externalise because they felt very normal” (P7).

When the AV and “healthy self” were not easily differentiated, participants became caught up in their thoughts and were less able to tune into their needs. Moreover, some questioned what the externalised entity was and how they should relate to it:

“I didn't really know what it [the AV] was…was it a separate being? or something in my brain that was telling me to do something?” (P6).

Some participants continued to grapple with their conceptualisation of AN during recovery post treatment:

“Even now I still think ‘How can I want to do something that I don't want to do?’ It’s a weird concept, even 10 years down the line” (P4).

**2.**: **Making sense of the Anorexia**

Participants described how therapies drew on creative therapeutic exercises to separate the AN from their identity. These exercises were the mode through which externalising language was learned and applied.

***2a.***: ***Person-led, experience-near language***

Participants reflected on the language used to make sense of their experiences in treatment. Some participants felt that combative language authorised a repositioning of themselves against the AV which they felt “neutral” language would not have achieved. In this way, it felt “productive” to “fight against” the AV:

“You could never really say anything neutral or that you're working with it in any way. It has to be ‘me against you’ meaning, otherwise you're not recovering” (P4).

However, other participants highlighted the importance of “neutral language” for combative language evoked exhaustion, a sense of failure, and a “louder”, “more controlling” AV. Instead, they found it helpful to adopt a diffusing stance in response to the AV.

“It [combative language] made the eating disorder louder. A more compassionate voice towards the eating disorder was better for me. If I talked back to it, it became more of a conflicting argument and made the eating disorder want more control” (P6).

Participants emphasised how therapists and treatment teams should be attuned to the emotional effects of externalising language and to how their experience of the same language could be helpful or hindering at different stages of recovery. For example, one participant initially found it more helpful to “question their own thoughts”, rather than consider the influence of an external force. However, the way that they related to their experiences of AN changed later on in their recovery. Implicit in her communication is the effect of socialisation to dominant externalising language:

“At some point, it [externalisation] does make things slot together a bit. Rather than thinking ‘Oh, I’m crazy’, I now think ‘There was a force over me making me do that’. You start to think, ‘Oh, that makes a lot more sense’” (P4).

Participants emphasised how crucial it is that therapists and treatment teams are led by the individual in order that externalising language has personal resonance*.* This approach was contrasted with the use of externalisation in a generalised, leading and assumptive manner. Hence, participants raised the importance of initially developing a shared understanding of the externalised concept. For instance, one participant described how attributing her experiences to an external entity would have felt inconsistent with her lived experience. Instead, she preferred to label her thoughts as "eating disorder thoughts" and differentiate those from her "healthy thoughts". Consequently, she valued how the therapist was led by her own choice of language during the process of constructing a shared understanding of her experiences:

“I think that if he'd [therapist] gone straight in with, ‘Oh think of it as a separate person to yourself’, I just wouldn't have got on with it because…it [viewing AN as an external third entity] would have felt weird to me. So it was a helpful process to discuss what that concept [the eating disorder] meant for me” (P7).

***2b.***: ***Using creative means to express complex feelings***

Whilst externalising exercises were often helpful in creating distance between the individual and the anorexia, the process felt nuanced, complex, and confusing. Letter writing was a difficult but “powerful” and validating task requiring “honesty”, “vulnerability” and “reflective” capacity. This process was helpful in supporting some participants to consider the functions and effects of AN on their lives. However, others felt unable to engage in letter writing in a meaningful way because the exercise felt “trivial”, they did not have emotional or cognitive capacity, or they did not experience any separation between a sense of true/authentic or inherently well sense of self (most commonly referred to as one’s “healthy self”) and their internal dialogue around eating (the AV):

“I found the idea [writing to AN] quite cringe. So I never kind of took up that opportunity and I don't know that I would have found it helpful. As much as I can say that there's one side of me that’s that and there’s one side of me that’s the other, and there are different paths of thought, it [the AN] very much still feels like it was a part of me” (P8).

Some participants felt that further abstracting the AV through visual imagery was not meaningful, whereas others felt that a visual representation gave them something “tangible” to communicate their experiences. However, it was essential that the image came from the individual rather than given to them. For instance, one participant was told to visualise an image of the AV which invalidated their distress:

“She [therapist] was like ‘Oh, just picture a goblin on your shoulders’, And I was like, “I clearly don't, so... I feel like you don't understand. You have no idea how intense this is. The idea was a bit strange. It didn't really work with me. I thought it was quite infantile” (P5).

In contrast, being asked to draw their experience of the AV helped some participants to make greater sense of AN:

“She [therapist] got me to draw, me on one side and what anorexia looked like on the other side and how that energy looked […]. And I had to visualise where that voice came from and identify where I felt it in my body and head, where it would come and what it would say. That was a really important thing I did because then visually, I just processed it, and it helped me to understand” (P10).

Arriving at their own visual depictions of their experience of AN enabled participants to express themselves in a way which enhanced the therapist’s perceived understanding:

“I would imagine that the thoughts were coming from that person [participant’s visual image of AN]. It helps me to separate it and to realise what was that voice and what was my own healthy voice. It helped her to see what I was imagining when I was thinking about the voice” (P9).

However, some participants found it challenging to engage in visual imagery as the exercise did not resonate with their lived experience in which the AN was perceived as a part of them, rather than as an external entity:

“I didn't really know how to imagine it, because I didn't really imagine it as like a physical person. It was more kind of like the thoughts and the voice of it. I didn't really see it as like a separate person as such” (P12).

Whilst personification of the AV was not perceived as having therapeutic benefit to all participants, most participants were able to engage in therapist facilitated dialogue with the AV. This was perceived as being the most helpful and generalisable skill acquired in therapy that they used to sustain their recovery:

“You talk from the eating disorder and then you get in the other chair and you’re back to you. To be fair, it does feel sometimes like I have two voices in my head, a healthy me and the unhealthy me. So, I do find it quite helpful to legalise it as two people” (P12).

Letter writing to AN as a friend and enemy, and to ones’ future self, as well as drawing AN were considered by some participants to be helpful at the time, however they did not feel the need to repeat these exercises. Instead, what helped to sustain recovery was continued daily management of their internal dialogue which they had practiced through role play, chair work or externalising conversations:

"Some of the actual physical exercises in terms of writing to it and drawing it [...] at the stage I’m at in recovery [...] I feel like those are things that I've done and they were helpful but I don't need to do them again, whereas it's more the daily internal stuff that I do more frequently" (P3).

**3.**: **Feeling seen, or unseen as a person beyond the Anorexia**

Participants described how externalising language could have a harmful or helpful effect on their interactions with others, including with their therapist, treatment team, family, partners and friends.

***3a.***: ***Replacing trust in the Anorexia with trust in the therapist***

Participants described ambivalence about treatment. Feeling ‘understood”, “seen as a person” and experiencing “connection” were significant in building trust in the therapist and treatment team, and in turn, in an externalised conceptualisation of their internal experiences:

“I trust my therapist as a person. When I started to do it [externalisation], it was somebody that was consistent in my treatment. Being understood was definitely a big part of it. She seemed to understand exactly what was going on in my head and so because she understood it so well, it made me realise ‘Oh, I see, this is anorexia’” (P9).

Externalisation provided a “framework” and “common ground” to build a collaborative relationship, permitting the individual and therapist to stand together “on the same team” against the AV. Experiencing the therapist relate to them “as a person” through the use of externalising language had a positive impact on participants’ sense of selves and instilled hope that AN was impermanent. Implicit within participants’ narratives is how externalisation provided the linguistic scaffolding that enabled them to experience a sense of ‘self’ as a person beyond the AN. This process subsequently permitted a re-authoring of their personal identity which had been lost to the dominant ED identity:

“It [externalisation] allows me to see myself as a person and not the eating disorder. That made me feel that she [therapist] believed that I could get rid of it as well. Seeing that someone else sees that pushes you forward” (P12).

However, some participants discussed that therapists should be careful not to “over externalise” their experiences as they perceived the AV to be external yet simultaneously “a part of them”. Some participants described a demeaning experience within inpatient settings whereby externalising language was used in a way which contributed to them feeling overlooked and de-valued:

“Staff on the ward need to use it a lot less because the result is you feel belittled. It really annoyed me. If I didn't like a certain food, […] and the nurses would say ‘that's your eating disorder talking’” (P13).

Further, it was unhelpful for therapists and treatment teams to take an “aggressive”, “controlling” or “forceful” stance during externalising conversations as they evoked heightened emotions and caused participants to retreat into the ED:

“When people try and enforce things on me, things get a lot harder for them because I dig my heels in, and this is probably where I switch into anorexia mode” (P11).

It was more helpful for the therapist to use externalisation in a “subtle”, sensitive manner through engaging participants in curious, tentative conversations which explored the influence of AN on their lives, whilst acknowledging who they were as a person beyond AN and the complexity of their relationship with the AN. In the context of a positive therapeutic relationship, participants began to replace trust in the AV with trust in their therapist:

“I started to hear my therapists’ voice there as well saying what I should…what is the healthy response? I think as I started to trust her more, I started to listen to the anorexia voice less” (P9).

Participants experienced shame, guilt and fear of disappointing their therapist when they had “given in to the voice”. However, externalisation helped to mitigate difficult feelings in the therapeutic relationship as they could “blame” their actions on “the eating disorder”. Sharing their internal experiences with others could feel exposing and unsafe, however talking about the influence of an external entity provided a way of communicating which required less vulnerability, making interactions with their therapist feel less intense, attacking and threatening. Some conveyed how talking about a “practical” “manifestation” helped to keep distance in the therapeutic relationship which supported them to engage in treatment:

“When I'm talking about it as the eating disorder as in the external manifestation of it, it feels less personal to me. It's less emotionally connected to me […]. Whereas to tell someone “I feel this way” or “I did this behaviour” feels a lot more intense and scary. You don't necessarily always want to cross that boundary and lay that vulnerability. It’s kind of ‘It's happening over there’…So it's more about keeping someone else at arm's length” (P5).

***3b.***: ***Scaffolding supportive relationships***

Most participants conveyed how others’ use of externalising language early on in their recovery could cause conflict, disconnection and distance in their relationships with family and close others. Some participants implicitly conveyed how externalisation caused harm when used inappropriately, such as when others named and discerned what they saw as the AN and its effects rather than seeking to learn about the effects of the AN from the individual themselves. This left the participant feeling lonely and misunderstood:

“When I was still very unwell, it made me feel very alone and isolated because no one was understanding me and what was going on for me in my head. It used to really make me cross when my mum would say, ‘That's anorexia talking’ because I didn't know that that was the anorexia talking” (P2).

However later on, some participants felt that the conceptualisation of AN as an external illness or entity helped them to explain their experiences to family and close others in a way that enhanced empathic understanding. However, significant to feeling understood was that family and close others used the same language as participants and that they were sensitive to the functions of the ED within the context of the individual’s life. One participant related their family’s difficulty with engaging in family therapy to their family’s understanding of AN:

“They very much saw it as [participant’s name] is the one that’s ill or she’s got this person on her shoulder’ but in that sense, it didn't help them to be more open to seeing it [the AN] as a systemic issue” (P13).

It was important that family and close others understood the influence of AN on participants’ thoughts, feelings and behaviours. Some participants felt that family and close others struggled to grasp an externalised conceptualisation of their difficulties and that this impacted on their containment of emotion and led to relational ruptures. Other participants explained how viewing AN as an external force helped to alleviate relational strain through supporting family and close others to manage difficult emotions, and reducing their own shame and guilt related to the impact of their difficulties on others:

“It makes it easier for family and friends to understand that it's not you that's behaving in that way, you're not choosing to behave in the way that you are. Cause that's what a lot of people think about eating disorders - that it's a choice to do what you do” (P3).

However, it was unhelpful when family and close others attributed all of their communication to “the eating disorder”, or when they used language which evoked feelings of failure and distress. One participant explained how their friends’ use of adversarial metaphors contributed to their withdrawal from the relationship and their retreat into the ED:

“When I'm hearing it [externalising language] from my therapist, it's calm and reassuring. But with my friends, it's forceful…it's completely different, and that puts me more on edge. It makes me feel more of a failure if things don't go right” (P11).

**4.**: **Navigating a complex relationship with the Anorexia**

Given the complexity of their relationship with AN, therapist facilitated exploration of both (1) the influence of the AN on the person and (2) the influence of the person on the AN were needed for the individual to experience a sense of personal agency in externalising conversations.

***4a.***: ***A constant presence; to listen, or not to listen?***

Participants continued to feel in relationship with the externalised ED post-treatment and weight-restoration:

“It's been an ongoing cycle that I've been in all my life. So, it's about managing my illness, allowing me to still do the things that I have to do” (P11).

The metaphor of the AV created space to not only explore the effects of the ED on the person, but also the person’s influence over this voice. Exploring the individual’s influence over the AV granted them with a sense of choice over whether or not to listen to the voice of the AN:

“Being able to separate the two voices is one of the things that has helped me to stay well because I can say, ‘Okay, I'm not listening to... that’s the eating disorder voice’” (P8).

Some participants attributed belief that they “would never fully recover” to internal factors such as their “personality”, “genetics” and/or “brain structure”. However, the metaphor of the AV enabled participants to experience and maintain a sense of distance between their perception of “self” and “the ED”. This enabled them to manage its impact and “carry on” “living a normal life” alongside it:

“I know that I've got something that people don't have that has the potential to destroy me and my sanity, or I can try to be stronger than it and do what I can to work against it. It's almost like another voice that I'm trying to just leave behind or ignore. And the more you ignore it, the better you get at it” (P4).

In recovery, participants acknowledged how easily they could “slip back” “under the spell” of the AV. They reflected on missing the “protective” functions of their relationship with the AV during times of difficult emotion and relapse was conceptualised as “listening to the voice again. Hence, emphasis was placed on “not trusting” the AV; understanding when and how it tried to intervene during times of vulnerability was crucial in staying well:

“My relationship [with the AN] has become a lot more separated, not as close, I guess. Trying to see the eating disorder as a sort of different person, but not necessarily a friend. Or not someone that I should feel the need to go to, or trust as much as I have done before. So it's not changed that much, but at the same time, I've just become a lot more aware that it's not with me for the right reasons” (P6).

However, all of the participants expressed difficulty with taking accountability through a tendency to blame their actions on a “third person”. Some conveyed how externalisation gave but also took their perceived “power” away because the externalised ED entity became an “oppressive”, “omniscient figure” that was “impossible to stand against”. These participants considered whether it would be helpful to use language which evoked less fear:

“Tread carefully not to overdo it so it becomes an invincible, powerful thing that becomes an excuse…you're like, well, I don't have control because it's actually not me that…the eating disorder is telling me this so therefore, I'm not going to eat” (P13).

Implicit within these participants’ narratives was that one-way externalisation which only explored the influence of AN on the person led to reduced personal agency, feelings of loss, helplessness and hopelessness. For instance, one participant at an earlier stage of recovery described fear and anticipatory loss in their relationship to the externalised AN as they did not experience a sense of personal identity separate to that of the AN identity. Consequently, they did not experience any agency in terms of their own influence over AN:

“By externalising it, am I going to go through the grief period of loss because it's been a part of me for so long, and now that we are separating it and I'm moving away from it, well that’s the idea. Is that then going to kick off another relapse? Am I gonna be lost without it and want it back?” (P11).

In contrast, when externalisation was extended so that the individual noticed their influence over AN, greater agency was experienced in relation to the ED. Hence, two-way externalisation served to mitigate participants’ feelings of vulnerability in relation to AN and enhanced their ability to take accountability for their recovery. For instance, one participant discussed how her husband did not use externalising language and spoke to her directly about “making the right decisions”. Being spoken to as though she had authority over her eating behaviour empowered her to sustain recovery:

“You don’t have to be accountable for your actions when you have a…when it’s something you want to do in secret and you've got someone to blame, it goes hand in hand. Him holding me accountable for my own actions stops either of us blaming it on a third party” (P4).

***4b.***: ***Reclaiming a valued sense of self***

Participants described their attachment to AN; they did not know who they were without it. Hence, when the influence of the person on the AN was not explored, participants felt entrapped within their relationship to an overwhelming AN. Externalising conversations which were focussed on “strengthening” one’s valued sense of self were more empowering than solely focusing on eradicating AN from their lives:

“If you've been unwell for quite a long time, feeling like you're just losing the eating disorder can be quite daunting. But if you’re actually building something else up as well…it felt a bit more hopeful…this idea that inherently there is still a healthy self inside you, rather than just assume you’ve been consumed completely (P5)”.

Exploring and understanding the development and function of the individual’s relationship with the AV served to reduce AN’s “bargaining power” and increase compassionate self-understanding:

“Rather than me deciding to have it in my life, I’ve looked at it as a separate being that's tried to intervene and interrupt things. Therapy allowed me to become more self-aware, but also understand why the eating disorder’s there in the first place. Because I think that so much of it is about wanting to be happier. And I think I've just proved to myself that doing those behaviours isn't making me happier” (P6).

Emphasis was placed on reclaiming a valued sense of self that had been obscured by the AN identity. Externalising conversations which provided participants with the linguistic scaffolding and opportunity to consider “what they truly wanted” in life increased hope and motivation for a different way of living. The linguistic separation between a valued sense of self and the voice of AN made it easier to visualise the “freedoms” that may be experienced without its influence. Accordingly, reflection on their personal values and aspirations helped participants to become more in touch with themselves as individuals beyond AN, which in turn harnessed strength and determination to resist the AV:

“Exploring my values as a person was really helpful in strengthening my own identity and helping me to externalise the anorexia voice because anorexic values are not my values, so why am I listening to it” (P11).

In recovery, participants accentuated the significance of experiencing a sense of “worth” and “purpose” in terms of their personal identity and lives in order to feel willing to detach themselves from the AN identity. What helped participants to stay well was reminding themselves of the impact that AN had on their lives, and their prioritisation of important relationships and aspects of life over their relationship with the AV:

“I know it can ruin marriages, friendships…it's really powerful. Thinking of it as a separate thing, I can think ‘Well, you're not gonna ruin my marriage’, ‘You're not gonna ruin my career’” (P4).

Therapeutic conversations which allowed for exploration of the person's influence over their life enabled the reclamation of a valued sense of self which was built on self-reflected values, hopes and dreams. Alongside this, externalising AN made room for the person to reflect on how the AN was getting in the way of the sort of life they hoped for and who they understood themselves to be.

## Discussion

Participants described a journey in terms of their experience of externalisation in recovery from AN. This journey is depicted through each main theme which is discussed in relation to theory, research and clinical implications. These findings from participants' experiences of externalisation were in the context of NICE recommended interventions for AN.

When the AV and “healthy self” were not easily differentiated, participants became caught up in their thoughts and were less able to tune into their needs. Moreover, some questioned what the externalised entity was and how they should relate to it:

“I didn't really know what it [the AV] was…was it a separate being? or something in my brain that was telling me to do something?” (P6).

Some participants continued to grapple with their conceptualisation of AN during recovery post treatment:

“Even now I still think ‘How can I want to do something that I don't want to do?’ It’s a weird concept, even 10 years down the line” (P4).

### Separating the Anorexia from the self

#### The Anorexia as one part of a multi-voiced self rather than an external third entity

ED-focussed therapies that sought to separate the person from the problem through psychoeducation, including explanations that AN is not part of the person was unhelpful for participants in the early stages of treatment. This explanation did not fit with their lived experience. Consequently, it was experienced as a rupture in the therapeutic alliance and a sense that therapists did not understand, or empathise with their experiences. What was more helpful later in treatment for some participants was a shared sense that AN was ‘a part of the self’ rather than all of the self; a part that was somewhat split from the self that they could therefore shift their relationship with to reclaim a sense of identity outside of the AN. However, other participants continued to grapple with how they should make sense of the AN throughout and beyond treatment; this contributed to their felt sense that the AN could return at any time and thus, their perceived vulnerability to relapse. They questioned whether the voice of AN was “a separate being”, “a part of themselves”, or “something in their brain”. The finding that making sense of AN is an ambiguous process is consistent with research which demonstrates that AN is difficult for individuals to make sense of, with some individuals holding dual concepts of AN as both a part of themselves and as separate from their identity [33]. The findings provide further insight by illustrating how an externalised conceptualisation of ones’ internal dialogue can be difficult to comprehend, particularly at the start of treatment. However, through their exposure to externalising language throughout treatment, participants began to engage with the notion of an internal ‘anorexia voice’ (the AV) which was to some extent split from the self. Over time and in ‘the context of positive therapeutic relationships, engagement with this concept aided engagement in treatment, suggesting that externalisation is an important component of treatment for AN. However, for some participants a sense of ambiguity in terms of not knowing what ‘the AN’ is and how they should perceive it can remain beyond treatment.

The findings provide insight into the complexity of the self in relation to the AV. Dialogical Self Theory is based on theories of self-multiplicity and assumes that the mind contains multiple ‘I-positions’ which can agree or oppose one another [46]. The internal dialogue between the different positions is important for the development and maintenance of personal identity [47]. The multi-voiced self can become dysfunctional if the person has a limited number of self-positions, they are not aware of other positions, or they are aware of competing positions but are not able to reach an overarching point of view to reveal a new position [47]. From this perspective, the externalised AN (the AV) is one I-position and ED recovery is thought to be related to changes in the dialogical self, such as the strengthening of adaptive internal voices to counteract the AV [48]. The findings illustrate how externalisation can help individuals to obtain distance from the AV, as well as access and strengthen an alternative I-position through separating ‘the AV’ from a perceived ‘healthy’ sense of self which is nurtured to reduce the AVs influence. Moreover, the findings are consistent with research demonstrating that at the onset of AN, individuals experience the I-position taken up by AN as positive and functional, however, as the ED progresses, individuals perceive the AV to be a controlling, critical, dominant and bullying external force [49–53]. The findings demonstrate how externalisation can support this shift in perspective by facilitating the individual’s reflection on the effects of the AV on their lives and relationships. Moreover, the theory of self-multiplicity and therefore the understanding that the AV may represent one of many parts of the self may serve to reduce ambiguity among individuals who struggle to make sense of the AN entity throughout and beyond treatment. This understanding may be experienced as less perplexing and ambiguous. Therefore, it may be more containing for the experiencing individual.

#### Locating Anorexia and people’s relationships to their bodies and food within their social-cultural contexts

Treatment socialised participants to terms such as the ‘healthy self’ versus ‘the anorexia voice’ which helped them to engage in treatment. Nonetheless, participants found themselves within a complex social reality whereby differentiating between the perceived ‘healthy’ and ‘anorexia’ parts of the self was challenging and nuanced due to the influence of dominant diet-related discourses. The participant narratives highlight how evidence-based ED interventions that focus on externalisation of the problem in the absence of problem deconstruction and repositioning means that dominant discourse (e.g., diet culture) are left intact and the “grey area” between the self and AN becomes more uncertain and difficult to discern.

A significant component of externalising conversations is that considerations beyond the individual can be taken into account [4, 54]). From a narrative stance, attention is always paid to the context in which problem-dominated stories exist including the ideas and beliefs that maintain their influence. Thus, an impetus of narrative therapy is to call into question (i.e., deconstruct) the dominant stories we may subscribe to, as well as the dominant forms of discourse used to construct these stories [25, 26, 55, 56]. Therefore, the therapist takes both a deconstructionist and constitutionalist position in order to empower clients to deconstruct the sense they make of their lives, the language practices they use, and the power relationships in which they find themselves [3]. Accordingly, externalising conversations are intended as a tool to invite people to identify and unpack the cultural knowledges they live by, as well as the taken-for-granted dominant language/discourse intertwined with one’s culture [56, 57].

The positioning of the clinician in this way within NICE recommended therapies for AN would serve to acknowledge the client’s internalisation of societal standards which have led to their understandable aspiring to valued ideals. Contextualising AN by locating people’s relationships to food and their body within their social contexts may serve to contain distress through making sense of the client’s experiences, including why it may be difficult to discern the ‘healthy’ and ‘anorexia’ parts of the self. Deconstructive inspired conversations and questions would enable the client and therapist to expose and unpack the social-cultural assumptions supporting dominant ways of thinking such as those that serve to maintain an over-evaluation of weight, shape and their control in the judgement of one’s self-worth.

#### Relational metaphors versus adversarial and medical discourses to externalise the Anorexia

A dominant way that externalisation is practiced in the NICE approved interventions for AN that were experienced by individuals in this study is through the metaphors of ‘AN as an illness’ which is experienced as an internal ‘voice’ versus ‘the healthy self’. These metaphors are built on illness discourse which demarcate two identity positions of illness (‘AV’) and recovery (‘healthy self’) which may also be understood as an ‘ideal’, ‘true’ or ‘authentic’ self. The participant narratives depict how the discursive materials available to individuals experiencing AN are limited to a dualistic medical discourse which restricts the terms of speaking about the experience of AN to the dualistic identity positions of 'sick' or 'recovered'. Individuals with lived experience of AN do not resonate with recovery discourse in which recovery is defined as an end-point where the individual lives in the absence of the illness [6, 7]. The participant narratives elucidate the challenges faced by individuals in their attempts to find resonating language to depict their complex social reality where AN is experienced as both a part of them as well as an entity within which they live in relationship to. Metaphors borrowed from medical discourse may limit the scope of therapeutic conversations built on participants' own experience-near metaphors that capture their complex social reality and have scope for externalisation that is more relational. The latter would enable the exploration of changes in the individual's relationship with AN over time, including the ways that they have reclaimed their life and identity from the AN [6, 7]. In contrast, when metaphors are borrowed from medical discourse, externalising conversations may focus less on the latter and more on eradicating the AN from the person's life. Both this study and existing research has shown that this can lead to exhaustion when an eradication of one’s relationship with AN does not eventuate [6, 7].

The metaphors that are selected to externalise AN will shape the individual's engagement and experiences of externalisation, and thus they will have influence over whether this practice is helpful or hindering to the individual's reclamation of identity from AN. An over-reliance on medical discourse may interfere with people's ability to make sense of their experiences in a meaningful way that enables them to continue to live in the face of eating-related difficulties. Reconceptualizing AN as an identity journey is thought to provide the scaffolding for individuals to (re)connect with important senses of identity in the face of suffering [6]. Prioritising the individual's own language forms in externalising conversations and relational metaphors may be better able to navigate people's complex social realities and give greater scope to richly explore the person's shifting relationship with AN, including ways that they have reclaimed their life and identity from AN.

### Making sense of the Anorexia

#### Individual led, experience-near language to externalise the Anorexia

The findings underscore the importance of being individual-led when making sense of the AV. The intended effects of externalisation are diminished in the absence of a context in which the client is viewed as the expert on their life [58]. Hence, curiosity and willingness to ask questions to which the therapist does not know the answer are essential narrative therapy principles that underpin personally meaningful externalising conversations [28]. It was hindering to participant’s engagement when therapists and treatment teams enforced their own conceptualisations of AN onto the individual’s experiences. Hence, the findings accentuate the importance of using language that is congruent with the individual’s lived experience, acknowledging that unique individuals do not have identical experiences of AN. This aligns with research which suggests that verbal synchrony between patients and their therapists contributes to positive treatment outcomes for AN [59].

Relatedly, the findings stress the importance of paying attention to individuality, for each participants’ experience of externalising practices was unique, and the same practice could be helpful or hindering at different stages of recovery. White asserts that therapists should continually consult with people about the perceived effects of their therapeutic work to ensure it remains meaningful, relevant and helpful [56]. Thus, regularly reviewing the effects of externalising practices with individuals in treatment may help to promote their positive effects such as that people feel empowered rather that oppressed in relation to the AN.

#### Cool versus hot engagement with the Anorexia

Participants appreciated the therapist taking up a compassionate, non-coercive and neutral stance during externalising conversations. Within the context of a previous high level of intervention for a persistent problem, rather than directly attempting to vanquish problems from people’s lives, it can be conducive to start with creating a reflective space through externalising conversations [29]. Participants valued externalising practices which acknowledged their attachment to AN over a ‘forceful’ approach in which the therapeutic focus was eradicating AN. This may be related to the bond between the individual and AV which is thought to explain ambivalence to change [50, 60]. The latter studies advocate that therapists penetrate the tie between the individual and AV, whilst acknowledging the AV’s hold. White emphasises that early externalising conversations should not focus on encouraging the individual to engage in a struggle with the problem, but rather to develop a shared understanding of the problem’s character, operations, activities, and purpose [29]. White termed this ‘cool engagement’ and discussed how this posture can alleviate vulnerability and distress in relation to the voice of a problem, in comparison to a ‘hot engagement’ which promotes direct confrontation with it. Therefore, in contrast to taking a directive, confrontational approach to the AV, it may be beneficial for therapists to create a reflective space for the individual to explore, understand and revise their relationship with the AV within the containment of the therapeutic relationship. These conversations may orient the client to adopt the intended ‘investigative reporter’ stance which can support the development of a more detached relationship with the voice of AN. In doing so, the client and therapist may collaboratively co-construct an ‘expose’ of the AN’s operations, activities, agenda, reinforcers, and importantly, map the AN’s effects on the person’s life and relationships.

#### Combative language versus a defusing stance in response to the Anorexia

Participants expressed particular familiarity with combative language in evidence-based treatments for AN. Metaphors are significant in externalising conversations; they are borrowed from discourses that contribute to specific understandings of life and identity and therefore shape an individual’s life and opportunities for action in relation to a problem [29]. Adversarial metaphors such as ‘fighting’ can contribute to feelings of defeat, failure, fatigue, overwhelm and reduced personal agency [29]. Furthermore, totalising the problem (dualistically defining it in totally negative terms) can obscure its broader context and invalidate what people give value to [29]. Hence, White did not intend for the position taken in relation to the problem to be either for, or against. Instead, the individual is invited to take a position that creates space for them to begin to reclaim their life from its effects. White suggests that to support individuals to revise their relationship to a problem, therapists should prioritise the use of metaphors which do not have adverse effects [29]. Accordingly, reclamation metaphors (e.g., ‘getting one’s life back from the problem’) should be prioritised over competition metaphors (e.g., ‘beating the problem’). Externalising the AV by commanding and gaining control over it is considered an important aspect of recovery in NICE recommended treatments for AN [8, 61]. Thus, it is common that metaphors privileged in treatment place people in a ‘battle’ or ‘fight’ ‘against AN’. However, ‘fighting’ the AV has been associated with more severe ED symptoms and distress [62, 63]. Therefore, researchers have begun to question which ways of responding to the AV are helpful versus problematic, suggesting that ‘compassionate assertiveness’ may be a more helpful response [48].

Combative language increased motivation to resist the AV for some participants, whereas for others, it activated their threat response, increasing the AVs dominance and the individuals’ submission. For these individuals, compassionate assertiveness and distancing from rather than arguing with the AV was more helpful. The findings are consistent with research in psychosis which suggests that aggressive counter-responding can stimulate threat-focused affective systems and heighten attention towards voices [64, 65]. Difficulty tolerating negative emotion is a trigger for engaging with the AV [66]. Therefore, Kater discusses the therapeutic benefit of using Acceptance and Commitment Therapy (ACT), stating that using hard data to argue with ED thoughts is not helpful in managing obsessive thoughts and preoccupation [67]. The findings support the aforementioned studies and suggest that individuals who experience increased preoccupation and distress on attempting to ‘fight’ the AV may find it more helpful to adopt a defusing stance. Encouraging individuals to adopt a defusing stance in relation to the AV may foster a less intense engagement with the AN. In doing so, the therapist may reduce the risk that the experiencing individual develops a fortress-type mentality that maintains the dominant problem-saturated AN identity story.

### Feeling seen as a person beyond the Anorexia

#### Revising one’s relationship with the Anorexia within the containment of the therapeutic relationship

The findings indicate that externalising practices are most helpful when they allow an individual to feel ‘seen as a person’. In narrative therapy, externalising conversations are intended to make it possible for people to separate their sense of identity from the problem-saturated accounts of themselves [68]. In this respect, externalisation is a powerful experience which permits scope for the individual to see themselves in ways that have been obscured by the problem-saturated AN identity. Clients consider the care relationship to be a meaningful contributor to recovery [69, 70], feeling treated as a ‘whole person’ and having a ‘real relationship’ with the therapist are regarded as significant by individuals experiencing AN [71]. The findings demonstrate how careful and thoughtful externalising practices can positively impact on an individual’s self-concept through enabling them to feel realised as a person by their therapist, rather than totalised as a ‘disorder’. This finding is consistent with research which concluded that the individual’s visual of themselves is expanded through them feeling treated as a person who is more than AN by their therapist [72]. In this way, externalising AN within the context of the therapeutic relationship invites the individual to think of themselves as people who are in relationship with a problem, rather than as problem-people (e.g., ‘an anorexic’). In turn, space is created for seeing the AN in new ways, opening possibility for developing a different relationship with the AN.

The findings support research which suggests that externalising the AV can provide a common language for therapist and client to work collaboratively despite the experience of ambivalence [73]. They also provide further insight by demonstrating that individuals need to develop trust in their therapist to become open to an externalised conceptualisation of their internal experiences. Individuals with AN deem a sense of connectedness between themselves and their therapist to be important for them to engage in adaptive relational processes, for instance self-disclosure [70]. Developing a shared understanding of the AV enabled participants to experience connection with their therapist, which in turn aided self-disclosure through mitigating the experience of shame in the relationship. In the context of a trusting therapeutic relationship, individuals internalised their therapists’ voice and drew on it to respond to the AV. In narrative therapy, the therapists’ position taken in relation to their client, and the relationship between them are considered fundamental in bringing about positive change [58]. Therefore, externalisation is thought to be therapeutically powerful because it reflects the quality of a relationship, rather than a technique [58]. The findings illustrate how thoughtful and skilfully practiced externalisation can aid the development of a positive therapeutic relationship in which the individual feels supported to revise their relationship with the AV within the containment of this alliance.

#### Privileging the voice of the experiencing individual when making sense of the Anorexia

Externalisation of AN as an external illness or entity helped to mitigate against family and close other’s perception that EDs are a ‘choice’, which in turn supported participants to maintain their important relationships. The findings are consistent with research demonstrating that caregivers can find it helpful to perceive AN as a separate entity as it enables them to attribute negative feelings to the ED rather than the individual [74]. However, they also provide new insight by demonstrating that when family and close others use language which is not congruent with the individuals’ understanding of their experience, or when it has adverse emotional effects, externalisation can negatively impact on relationships and recovery. White and Epston [26] emphasised that the externalised problem definition should be mutually acceptable. However, the FBT manual suggests picking a metaphor that works best for parents [13, 14]. The findings suggest it is crucial that family and close others use the individual’s own experience-near language to describe their difficulties, including their experience-near naming of the problem. Further, that they should be careful not to over-externalise the individual’s experiences as this may contribute to relational ruptures. For the majority of participants, the AN was experienced as a part of the self that they were in relationship with; this part manifested as an internal voice (the AV). Hence, encouraging the individual to adopt the view that the AN is an external third entity may invalidate the individual’s lived experience and implicitly invite them to take a total position in relation to the AN (i.e., all against it). A position that is total will contribute to anxiety and apprehension to take steps towards resisting the influence of AN. Instead, individuals wanted to make meaning out of their experiences and make sense of the AV’s development, purpose and role in their lives rather than dismiss it as a problem to vanquish.

When others inform the experiencing individual what is or is not ‘the ED/AN talking’, the individual is not positioned as the primary author of their own life. Instead, others take over expert status and the individual can become disempowered within the interaction. This practice of externalisation is one that has been taken up in many of the NICE approved therapeutic practices, however it is a divergence from the spirit of narrative therapy in which the individual experiencing a problem is positioned as the expert in their life and the author of their life story. Hence, the participant narratives depict the harmful effects of externalisation when it is removed from its core narrative therapy principles. The life story of the experiencing individual, being socially constructed within an interpersonal context, will be inevitably shaped by their interactions with others. When the individual is not invited to take a collaborative co-authoring consultative position there is risk that the person becomes totalised by dominant discursive language used in treatments for AN. The practice of externalisation as originally proposed in narrative therapy is that the experiencing person is the expert of their life, including in discerning what is and is not the AN [31]. Therefore, the participant narratives depict how important it is to privilege the voice of the experiencing individual in providing an account of their experience in all its complexities, rather than for others to assume what is, or is not ‘the anorexia talking’. Within the context of externalising conversations in narrative therapy, White [56] refers to an “insurrection of subjugated knowledges”, an opening that will allow the individual to construct the story of their lives in terms other than those dictated by the dominant narrative which feeds the problem. In therapies for AN, this would require the therapist and family/close others to privilege tentative curiosity, listening over questioning, and to question in a way that help clients to observe how their lives are actively constructed, rather than passively recounted and given.

Whilst externalising practices disarm internalising discourses that seek blame for the problem’s existence [28], making sense of the externalised problem and detailing its history is an important task from a narrative perspective [4]. The findings elucidate the risk that conceptualising AN as an external illness or third-entity might detract from psychological formulation which may contribute to family member understanding that the problem resides within the individual. Positive, helpful experiences of personal relationships are significant in AN recovery [75]. White advocated that the development of a shared understanding of the externalised problem within the context of the individual’s life experiences can assist their support network to be more containing and supportive of their needs and difficulties [58]. Participants reflected on the function of their relationship to the AV in terms of helping them to feel ‘safe’, ‘comforted’ and ‘protected’ from the emotions that arose from life stressors, transitions, interpersonal relationships and sociocultural pressures. They conveyed how it was important that their support network is understanding of the functions of the AN within the context of their life and relationships. Hence, it is important that others’ conceptualisations of AN acknowledge the psychological, emotional and social-cultural factors which contribute to its development and maintenance (i.e., AN should not be removed from the individual’s developmental history and social-cultural context).

### Navigating a complex relationship with the Anorexia

#### Exploring and deconstructing the experiencing individual’s relationship with the Anorexia

Whilst individuals feared the externalised AN and felt controlled by it, they also felt attached to it, daunted by the loss of it and missed it in recovery. Individuals describe their ED as ‘a life jacket’ that provides control, isolation, security, identity, and a tool for emotion regulation and avoidance [35, 76, 77]. Emphasising the negatives of AN without exploring its functions fails to acknowledge the meaning of ED behaviours, invalidates the individual, and neglects opportunity for finding alternative mechanisms through which needs can be met [38]. Rather, learning what an individual gives value to in life can help to build the foundation for collaboration [31]. Exploring and understanding ones’ relationship with the AV through the use of externalising practices which encouraged reflection on the AV’s function and effects enabled some participants to process their thoughts, feelings and experiences in relation to the AV and subsequently attempt to meet its perceived functions through alternative means. These therapeutic conversations aided the development of self-understanding and self-compassion which served to reduce enmeshment with the AV. In contrast, using externalising language to emphasise attempts to vanquish AN without acknowledging its role within the individual’s life served to increase resistance. These narratives align with research which has articulated how relational metaphors as oppose to illness meta-narrative that totalises AN on entirely negative terms enable individuals to linguistically separate their identity from the ED, while authoring their experiences as an enduring struggle that has shaped their identity [6].

The findings are consistent with research which conceptualises EDs as attachment-relationships [78]. Research has questioned whether the individual-AV relationship is reflective of early attachments and interpersonal ways of relating [48]. It has been suggested that exploring the individual-AV relationship may help to resolve relational patterns and attachment-related issues which maintain AN [60]. The findings demonstrate how externalisation can aid this process by facilitating the development of a relational understanding of AN. For instance, letter writing and chair work opened space for participants to speak to the voice of the AN that they experienced themselves as being in relationship with. However, given the attachment themes and interpersonal patterns within participants’ narratives in relation to the AV and therapeutic relationship, it may be beneficial to utilise externalisation to explore the individual-AV relationship in the context of attachment-related issues and the clients’ interpersonal patterns. Emerging research demonstrates the importance of utilising therapeutic approaches that address relational trauma with individuals who experience an AV [79]. Exploring the influence of early relationships and childhood experiences on the development of the AV may help to provide individuals with a greater sense of understanding in relation to their experience of AN and how to make sense of this entity. Here, the use of relational metaphors to characterise the individual's experience of AN may provide individuals with a discursive tool to mediate complex meanings and social realities. In doing so, relational metaphors may offer individuals an alternative context to author their narratives from the dualism of AN discourse in which their experience of eating-related difficulties is either present or gone, as well as removed from their developmental history and social-cultural context.

An important part of narrative therapy is deconstruction of the problem story that explores the history of dominant problematic identity stories to understand the context within which these were constructed, including who was there [3]. This process enables the individual to revisit these experiences through a different lens to re-author narratives that more comprehensively understand the development of these problematic storylines as well as the identity narratives that were lost in these contexts of a person’s history. The way externalisation has been taken up in many of the NICE approved interventions for AN is that externalisation has been utilised as a technique and taken out of the full context within which it was intended. The participant narratives reflect both the strengths of externalisation in these practices as well as some of the gaps and more problematic experiences that have eventuated through separating externalisation from the broader narrative practice in which it originated. This means that key assumptions and elements of the narrative practice of externalising have been omitted and neglected, for instance deconstruction of the problem, experience-near naming and the spirit of narrative therapy where the person is positioned as the expert of their life.

#### Enhancing the experiencing individual’s personal agency in relationship to the Anorexia

The narratives depict an interwoven relationship between vulnerability and accountability in relation to externalisation. They are consistent with research which depicts the experience of AN as being entrapped in a toxic, enmeshed relationship in which the self is shared with AN [50, 80]. Internal ED dialogue reflecting an ‘abusive relationship’ predicts ED severity, suggesting that in order to enhance personal agency, the connection between negative appraisals of the ‘abused self’ and the abusive voice of the ED must be alleviated [81]. Externalising the problem is intended to have the effect of ‘freeing’ the person to act independently of the problem [26]. Some participants questioned whether externalising AN as a powerful third-entity gave the AV more authority and reduced their sense of agency. Referral to the ED as a separate entity, as though it has a life of its own is a common discursive phenomenon between healthcare professionals and service-users [82]. A ‘stronger AV’ (i.e., with higher levels of voice power, omnipotence, entrapment and defeated response) is associated with increased ED severity and duration [63]. Therefore, certain appraisals of the AV may hinder recovery by further exacerbating identification with an abused self, increasing distress and feelings of entrapment. Individuals with lived experience of AN regard empowerment consisting of taking responsibility and control leading to confidence, agency, resilience, autonomy and independence to be significant in recovery [83]. Therefore, therapists and treatment teams should be cautious of using language which empowers rather than disempowers individuals in relation to their experience of eating-related difficulties.

Participants emphasised their difficulties with taking accountability and responsibility for their eating-related behaviours due to a tendency to blame their actions on a third-entity. The narrative approach attempts to decouple the effects of shame and blame from the act of taking responsibility for one’s problems [4]. From this perspective, problems can be seen as inviting particular ways of being and space is created for the person to choose a preferred way of responding to the problem. Therefore, externalising conversations are intended to allow space for clients to consider whether they may wish to decline invitations to respond to the influence of the problem. In order to support individuals to feel more empowered in treatments for AN, the therapist could be tentative with their choice of language such as that the individual is supported to recognise that they have control over how they choose to respond to the influence of the AN.

Implicit within participants' narratives was that dominant discourses within treatment for AN such as those that emphasise combative metaphors in relationship to an external third-entity can risk the problem becoming so overwhelming and insurmountable that the individual loses a sense of personal agency to act on their own behalf in the face of the eating-related difficulties. When externalisation focuses on one side of relative influence questioning and only explores the influence of AN on the person rather than also exploring the person’s influence on AN, the individual is disempowered in relation to the eating disorder [31]. In narrative therapy, externalising conversations are intended to undermine the sense of failure that may have developed for individuals in response to the continuing existence of the problem despite attempts to resolve it [4]. They are also intended to open up new possibilities for people to take action to reclaim their lives and relationships from the problem and its influence [4]. Externalising practices were most powerful when they created opportunities for dialogue about the influence of the eating disorder on the person’s life and relationships, as well as the persons influence over the eating disorder. Externalising was less meaningful when it was simply used as a ‘technique’ and removed from the philosophical world view of narrative therapy as it only served to create opportunities for monologue about the problem (e.g., the individual must ‘fight the AN’).

The findings highlight the importance of tracking the history of the problem and in doing so, mapping the person’s own influence in the life of the AN in addition to mapping the influence of the AN on the person’s life (relative influence questioning). This two-staged process would afford the therapist and client to arrive at an understanding that the client’s relationship with AN is non-static and may lead to the discovery of unique-outcomes. Inquiring about unique outcomes is an interview technique to help clients internalise personal agency and develop a self-narrative in which they view themselves as powerful [29]. Questions that bring forth information that challenges the dominant problem-saturated AN identity story and assist individuals in discovering resources, skills and competences that have not been realised under the influence of the AN may support individuals to take accountability and responsibility for how they choose to respond to the AN. In attempt to empower individuals in therapies for AN, the therapist could ask clients about particular instances in which they client have not been oppressed by AN, these unique outcomes may include times when AN was not present in their lives (i.e., life before AN) or times where they had greater influence over AN. Subsequently, the client could be invited to link the unique outcome to past events and extend the story into the future. In doing so, the client redescribes themselves and their relationship to AN in light of these exceptional events, forming alternative stories that are less problem-saturated and a preferred self-narrative in which the self is viewed as more powerful than the AN. These conversations could represent new opportunities to resist, rather than surrender to the influence of AN.

Furthermore, from a narrative perspective, externalising conversations can also take place with regards to preferred qualities (e.g., determination) [54]. Hence, it is possible for the therapist to reflexively inquire about how this quality came to exist, its effects, what it says about the person, what other qualities it is linked to, and where it is most/least prominent in the person’s life. This process within therapies for AN would support the person to experience their personal qualities as more meaningful and relevant in their life, which in turn may foster personal empowerment and lead to a greater sense of personal agency to resist the influence of AN. Additionally, from a narrative perspective, externalising the client and family’s experience of success is another externalising practice to support them to internalise their own competence [84]. Thus, when the client and their support system experience success in terms of their resistance to the influence of AN, this success could be externalised to assist them in the process of internalising personal agency. For instance, in discussing how the success occurred, the client and family could be encouraged to articulate qualities within themselves that have assisted them in overcoming AN’s influence. This process would serve to counter the depersonalising of personal and/or familial successes in their resistance to the influence of AN and thus reduce feelings of oppression and internal defectiveness.

#### Identifying and connecting with a valued sense of self and preferred ways of living beyond the Anorexia identity

Externalising conversations that gave voice to participants’ values, purpose, commitments and aspirations versus those of the AV’s were significant in helping individuals to realise their valued sense of selves and preferred ways of living. White’s rationale for externalising conversations was to make it possible for people to experience an identity that is separate from the problem; to open possibilities for the pursual of what is personally important [29]. A common response to externalising conversations in narrative therapy is that they have the effect of enabling the person to get more in touch with other, less problem-dominated narratives that speak about who they are [54]. In the course of AN, it can become increasingly difficult for people to find an alternative identity [37]. Individuals often comprehend recovery as desirable yet ‘unattainable’ and ‘unimaginable’ [85] as they fear losing a major part of their identity [86]. When an individual's vision for something is "unimaginable", change is "unattainable" [85]. Hence, imparting hope can enhance therapeutic alliance and in turn improve outcomes [87]. The findings depict how externalising practices can increase hope by helping individuals to connect with a seemingly ‘unimaginable future self’ through not only feeling realised by others, but also by their selves. The creation of therapeutic space to consider alternative stories to the dominant AN one shed light on other aspects of the person’s life that AN had been obscuring. Consequently, reflective conversations that open space for an alternative narrative identity to develop and be thickened may increase people’s willingness to “let go” of AN.

Developing therapeutic solutions within the narrative frame involves opening space for the authoring of alternative stories to those which have been marginalised by the dominant oppressive narrative which maintains the problem [3]. Separating the individual from the problem within therapies for AN appeared to open up possibilities for individuals to take control of their own lives through helping them to connect with preferred stories of living and identity. The findings highlight how important it is that these therapies allow sufficient time and space for the process of therapeutic reauthoring which would allow the individual to understand that their life and identity are multi-storied. Ensuring that there is a balance in focus on challenging the AN alongside the exploration of the person’s other stories of self would help to trace the social and relational history of valued aspects of life which may in turn support expressions of life that are not in harmony with the dominant AN identity story.

Lastly, distancing one’s self from the AV was a practice that participants used to minimise the AV’s influence on their life post-treatment and weight-restoration. Individuals who have been discharged from treatment after reaching a healthy weight describe AN recovery as an on-going process and emphasise the importance of psychological change (e.g., motivation and belief in the capacity to change) in sustaining recovery and managing relapse risk [88]. The findings suggest that externalisation can aid these processes of psychological change. An externalising attitude in narrative therapy has a powerful deconstructive effect; it positions the therapist to interact differently with people than they would if they saw them as in possession of intrinsic characterological problems [4]. The participant narratives regarding their relationship with AN post-treatment reflect their internalisation of the therapist’s externalising attitude in therapies for AN. Their internalisation of this stance towards the problem provided participants with a sense of agency over how much influence they allowed the AV to have on their lives. In turn, their internalisation of an externalising stance permitted space and authority to resist the consumption of their identity to the AN.

### Strengths, limitations and further research

Holding ‘insider research’ status has both advantages and disadvantages [89]. An advantage relevant to this study was the potential for a greater level of trust between participants and researcher [89]. This was observable throughout the interview process whereby the first author perceived a strong connection with participants. Consequently, the interviews were long in duration due to the rich and in-depth conversations about participants’ experiences, resulting in high-quality data.

However, the role of the insider-researcher in shaping knowledge production must be acknowledged rather than assuming that it offers a ‘correct’ way of viewing the population under study [90, 91]. The involvement of service-users in the development of the interview schedule may have mitigated against the latter by eliciting a wider range of interview questions and findings. Nonetheless, in line with an RTA approach, the first-author actively followed up what they interpreted as being meaningful to participants [92]. Additionally, the final interview question asked participants if there was anything that had not been asked which they felt was important to share. It is hoped that this question permitted participants the opportunity to share experiences and views which were not guided by the researchers. Nevertheless, the experiential knowledge of living with a condition provides relevance and credibility to research [93, 94]. Hence, future research focussed on externalisation would benefit from involving service-users in the development of the interview schedule.

Moreover, the sample was largely homogeneous and individuals who identify as male, or non-binary, as well as individuals from ethnic backgrounds other than white-British are underrepresented, thus replicating issues within the ED research field [42]. Both gender and ethnicity influence the experience of AN [95, 96]. Hence, eliciting insight into people’s experiences of externalisation in a more diverse sample may contribute to greater variation in the experience of externalisation.

Additionally, all participants were aged 20 and above and identified as being ‘in recovery’. Thus, future research should explore the experience of externalisation in treatment for AN among children, young people and families. Exploring experiences of externalisation among individuals who identify as ‘fully recovered’ or ‘not recovered’ may shed light on how externalisation supports full remission from AN.

Lastly, the findings of this study are limited to people's experiences of externalisation in the context of NICE approved treatments for AN. None of the participants had experienced narrative therapy and therefore more research is needed into people's experiences of externalisation in narrative therapy from which this practice was originally developed by White and Epston [26].

## Conclusions

Externalisation has been taken up in a number of NICE approved ED interventions. The findings of this study regarding the experiences of externalisation in those who have participated in these treatments underscore the importance of using externalisation in a person-centred manner, underpinned by core narrative therapy principles, to ensure that language empowers rather than disempowers individuals. Accordingly, the findings highlight the importance of working with individuals to develop a psychologically informed understanding of their experiences of eating-related difficulties within their social-cultural context, as well as to find and strengthen alternative identities that are preferred by the experiencing person and are strengthened through being aligned with their values, hopes and intentions for their life.

These findings underscore the importance of clinicians, treatment teams, and family members prioritising attunement to the individual’s lived experience, the language they use to author their experiences, and relational (rather than adversarial) metaphors in externalising conversations that have scope to author a complex social reality and make room to find meaningful stories that have been hidden by the dominant AN identity. More research is needed to explore the experience of externalisation in other therapeutic modalities that are under-researched, particularly narrative therapy from which externalisation was described more than 4 decades ago, including in working with individuals whose lives have become dominated by EDs.

## Supplementary Information

Additional file 1: Interview schedule.

## Data Availability

The datasets generated and analysed during the current study are confidential due to the need to protect the privacy of participants. Segments of anonymised data are provided within the manuscript. However, the full datasets generated and analysed during the current study are confidential due to the need to protect the privacy of participants.

## Abbreviations

UK: United Kingdom
AN: Anorexia Nervosa
ED: Eating disorder
AV: Anorexia voice/thoughts
FBT: Family based treatment for Anorexia Nervosa
FT-AN: Family therapy for Anorexia Nervosa
