# Supplementary material for: Experiences of externalisation in recovery from Anorexia Nervosa: a reflexive thematic analysis
Source: J Eat Disord. 2024 Oct 7;12:157. doi: 10.1186/s40337-024-01087-9 (PMC11460161; doi:10.1186/s40337-024-01087-9)
Supplement: Supplementary file 1 — Additional file 1: Interview schedule. [file 40337_2024_1087_MOESM1_ESM.pdf]

Additional file 1: Interview schedule

⇒ You told us in the questionnaire that you have engaged in psychological treatment for anorexia nervosa. We are really interested in your experiences of externalising an eating disorder throughout the recovery process in treatment.

⇒ We hope that by exploring people's experiences of externalisation, we can gain a better understanding of the ways in which externalising anorexia can help and hinder people's recovery from an eating disorder.

1. Before we talk about your experience of externalising anorexia in treatment, it would be helpful to hear where you feel you are in terms of recovery from an eating disorder?

2. How, when and by whom was the notion of externalising anorexia nervosa first introduced to you?

3. How was externalisation used in your treatment(s) for anorexia nervosa?
   how was it when

4. What language and metaphors were used to externalise your eating disorder?

   a. Who initiated this language/ use of those metaphors?

5. How did you experience externalising anorexia?

   a. What was your relationship to the externalised eating disorder?

6. How did externalising anorexia influence/ effect your relationship with other people, like your therapist or your family?

7. How did externalising anorexia influence/ effect your engagement in treatment?

8. What was helpful about externalising your eating disorder during your treatment and recovery, if anything?

9. What was unhelpful about externalising your eating disorder, if anything?

10. How has externalising anorexia impacted on your ability to stay well, positively or negatively?

11. What advice or recommendations would you give to professionals and/or family members about externalising anorexia?

⇒ Is there anything which has not been asked, or that we did not discuss that you would like to share about your experience of externalisation in recovery from an eating disorder?

⇒ Debriefing
